# Aqueous potentially ecotoxic metal(loid)s in a tropical mining-affected river system: sources and environmental and human health risks

**DOI:** 10.1007/s10653-025-02808-y

**Published:** 2025-10-17

**Authors:** John Kennedy Okewling, Matthew Eyre, Karen A. Hudson-Edwards

**Affiliations:** 1 Camborne School of Mines, University of Exeter, Penryn, TR10 9FE UK; 2 Environment and Sustainability Institute, University of Exeter, Penryn, TR10 9FE UK; 3 Ministry of Energy and Mineral Development, Mines Department, P.O. Box 9, Entebbe, Uganda

**Keywords:** Water contamination, Water quality assessment, Human health risk assessment, Potentially ecotoxic metal(loid)s (PEM), River Nyamwamba, Uganda

## Abstract

Water contamination poses threats not only to aquatic life but also to human health. Understanding mining-related potentially ecotoxic metal(loid) (PEM) contamination in a tropical environment is vital for regulation and management. The study aimed to examine the concentrations, sources, and environmental and human health risks of PEMs in tropical river systems. Using the mining-impacted River Nyamwamba in Southwestern Uganda as an exemplar case study, 19 water samples along the River Nyamwamba and its tributaries, and Lake George were collected. The samples were analysed with ICP-MS. Concentrations decreased in the order Co > Mn > Fe > Cu > Ni > Al > Zn > Mo > As > Pb > Cd = Cr. Cobalt, Mn, and Ni concentrations exceeded safe drinking water standards. Multivariate statistical analysis revealed that mining contributed to the presence of As, Co, Cu, Mn, Mo, Ni, and Zn. Pollution load index and potential ecological risk index indicated severe ecological risks. Health risk assessment showed that both carcinogenic and non-carcinogenic risks were posed to human health, with children being the most vulnerable. Up to 2 in 10 children and 3 in 100 adults were at risk of developing excess cancer from PEMs exposure in the river water. The study highlights the importance of preventing untreated aqueous and mine waste discharge into tropical river systems, and recommends that the local government sensitise the community and restrict the use of River Nyamwamba water in favour of other sources (shallow wells, boreholes, springs, and rainwater harvesting), while regularly monitoring water quality.

**Supplementary Information:**

The online version contains supplementary material available at 10.1007/s10653-025-02808-y.

## Introduction

Water is essential for the survival of living organisms (Ahmad et al., 2021; Belle et al., 2023; Nganje et al., 2020). Water quality continues to degrade globally due to contamination with potentially ecotoxic metal(loid)s (PEM), persistent organic pollutants (POPs), hydrocarbon contaminants, fertilisers, and pesticides (Dashtizadeh et al., 2019; Onen et al., 2023). This has led to the introduction of the sixth Sustainable Development Goal to ensure the availability and sustainable management of water for all (UN, 2015). Despite this, access to clean water has remained a significant global challenge (Ahmad et al., 2021; Dashtizadeh et al., 2019; Nganje et al., 2020) due to rapid global population growth, industrialisation, and urbanisation, with over 2.2 billion people lacking access (UN, 2023).

Potentially ecotoxic metal(loid)s are of particular concern for water quality because of their persistence, abundance, bioaccumulation, and biomagnification potential at the upper trophic levels (Ali et al., 2019; Belle et al., 2023; Elnabi et al., 2023; Tchounwou et al., 2014; Verma, 2020) as well as their capacity to hinder the survival, growth, and reproduction of organisms (Ali et al., 2019). Potentially ecotoxic metal(loid)s include Al, As, Cd, Co, Cr, Cu, Fe, Hg, Mn, Mo, Ni, Pb, Zn, etc. and are also known as trace elements because of their presence in low concentrations in various environmental components (Tchounwou et al., 2014; Verma, 2020). They have also been referred to as heavy metals (Ali & Khan, 2018; Macklin et al., 2006; Tchounwou et al., 2014), but due to the inclusion of elements like As, which is a metalloid rather than a metal, they are referred to in this study as PEMs due to their potential toxicity to the ecosystem. A PEM is defined as a naturally occurring element (metallic or metalloid) with a density greater than 5 g/cm^3^ (Elnabi et al., 2023; Macklin et al., 2006; Tchounwou et al., 2014; Verma, 2020). The concentrations of PEMs can be elevated relative to natural background concentrations by natural phenomena such as weathering, volcanic activities, earthquakes, hurricanes, tornadoes, flooding, tsunamis, soil erosion, sea salt, and natural forest fires (Tchounwou et al., 2014; Verma, 2020), and anthropogenic activities such as mining, industrial, agricultural, pharmaceutical, domestic waste disposal, and combustion of fossil fuels (Ali et al., 2019; Cao et al., 2023; Tchounwou et al., 2014). The degradation of water quality with PEMs can occur through the discharge (deliberate or accidental) of tailings (tailings storage facility failures, tailings blown by the wind, or tailings transported by floods) (Karna et al., 2021), leaching of PEMs from mineralised materials (Azevedo-Santos et al., 2021; Karna et al., 2021; Tchounwou et al., 2014), erosion of PEM-bearing soils (Tchounwou et al., 2014), and water flowing out of underground mine tunnels. Despite these being much-studied phenomena, PEM-contaminated water resources management remains complicated (Akhtar et al., 2021).

Aqueous PEM contamination can severely affect human health and living organisms (Ahmad et al., 2021; Akhtar et al., 2021; Nganje et al., 2020). Essential PEMs such as Cu, Fe, Mn, Ni, and Zn are important for living organisms, but they can become toxic beyond certain threshold concentrations depending on the dose and duration of exposure (Ali et al., 2019; Nganje et al., 2020; Verma, 2020). However, nonessential PEMs, including Pb, Cd, and As, do not have known biological functions in living organisms and are thus considered toxic even at very low concentrations (Ali et al., 2019; Elnabi et al., 2023; Hameed et al., 2020; Tchounwou et al., 2014; Verma, 2020). Human exposures through ingestion, inhalation, and dermal contact are the major pathways for PEM contaminants (Elnabi et al., 2023; Kaninga et al., 2020; Li et al., 2022; Tchounwou et al., 2014). Long-term exposures to PEMs can cause severe health issues affecting the respiratory, liver, kidney, cardiovascular, neurological, reproductive, gastrointestinal, and immune systems, potentially leading to chronic diseases, developmental problems, various cancers, and death (Elnabi et al., 2023; Hameed et al., 2020; Nganje et al., 2020; Tchounwou et al., 2014; Verma, 2020).

Mining activities can release PEMs into aqueous ecosystems through the discharge of mining effluents and mine waste solids (especially tailings) into water bodies (Belle et al., 2023). For example, the 2000 Novat-Rosu tailings dam failure in Romania released circa 100,000 cubic metres of contaminated water and 20,000 tonnes of mineral-rich solid waste into the River Novat (Bird et al., 2008). It has been suggested that the long-term discharge of mine waste into rivers poses risks to approximately 23 million people globally (Macklin et al., 2023). Using PEM-contaminated water for domestic purposes, recreational activities, and crop irrigation poses a major health risk for communities (Belle et al., 2023). Analysis of filtered aqueous PEMs can be useful in assessing the degree of contamination of an ecosystem (Alves et al., 2014). In recent years, environmental and human health risk assessment methodologies have been widely employed to evaluate the potential exposure risks derived from aqueous PEMs (Alves et al., 2014).

Several studies have evaluated mining-related PEM contamination with their associated environmental and human health impacts in temperate and arid river systems (Grimalt et al., 1999; Hudson-Edwards et al., 2001; Macklin et al., 2023). By contrast, many studies on tropical river water contamination, especially in Africa, have assessed PEM water contamination but lack incorporating a thorough investigation into the environmental and health risks associated with using the contaminated water for domestic and drinking purposes (Atibu et al., 2013; Mwesigye et al., 2016; Abraham & Susan, 2017Mwesigye et al., 2019). Understanding mining-related PEM contamination and the associated risk in such environments is vital for policy formulation to regulate and manage contamination, which underscores the need to analyse PEM contents in tropical river water and further predict ecological and human health impacts. To fill this research gap, the study aimed to use the mining-affected River Nyamwamba, a tropical river system in Uganda, as a representative case study to determine the suitability of the water quality for domestic and drinking purposes. The specific objectives of the study include (1) examining PEM concentrations, (2) using multivariate analysis to determine PEM sources, and (3) assessing the associated environmental and human health risks. The results will have implications for tropical areas with legacy, active, and/or abandoned mining sites, especially those involving metallic mining located near streams, rivers, lakes, and/or oceans.

## Materials and methods

### Area of study

The River Nyamwamba flows in the southwestern part of Uganda within the administrative district of Kasese. It is located in the eastern part of the slopes of the Rwenzori Mountains in the Albertine Rift region, rising in the alpine slopes of the Rwenzori Mountains at 4,546 m above sea level and flowing downslope for about 126 km to the confluence with the River Rukoki at 1,180 m above sea level (MWE, 2022). After the confluence, the River Nyamwamba flows into Lake George through the wetland on the lake’s northern shores.

The study area experiences a sub-humid equatorial climate consisting of a bimodal rainfall regime (MWE, 2020). Precipitation in the catchment primarily occurs during two seasons: shorter rains from March to May and longer rains from August to November; nonetheless, abrupt intense rainfall may occur in the dry seasons (Musonge et al., 2019). The Nyamwamba Catchment has an area of 260 km^2^ (MWE, 2022) and total annual rainfall ranging from 800 to 1600 mm (MWE, 2022) with a weighted annual average of 2,226 mm (SEA, 2010). Temperatures range from 0 °C at the peak to 30 °C at the plains (MWE, 2022). The annual mean discharge of River Nyamwamba was 33 m^3^/s in 2017 and is expected to be 52 m^3^/s by 2027 (Kimera & Tumwijukye, 2022).

Dense shrubs, wetlands, and cropland dominate the vegetation of the study area. Stratified natural vegetation zones of grasslands, woodlands, and even bamboo extend upstream of the study area in the hilly areas of the Rwenzori mountains. There has been an increase in the vegetation cover along the River Nyamwamba due to the abandonment of cultivation along the wetlands because of sensitisation and the destruction of gardens by the flooding of the river (KMC, 2023).

**Fig. 1:**
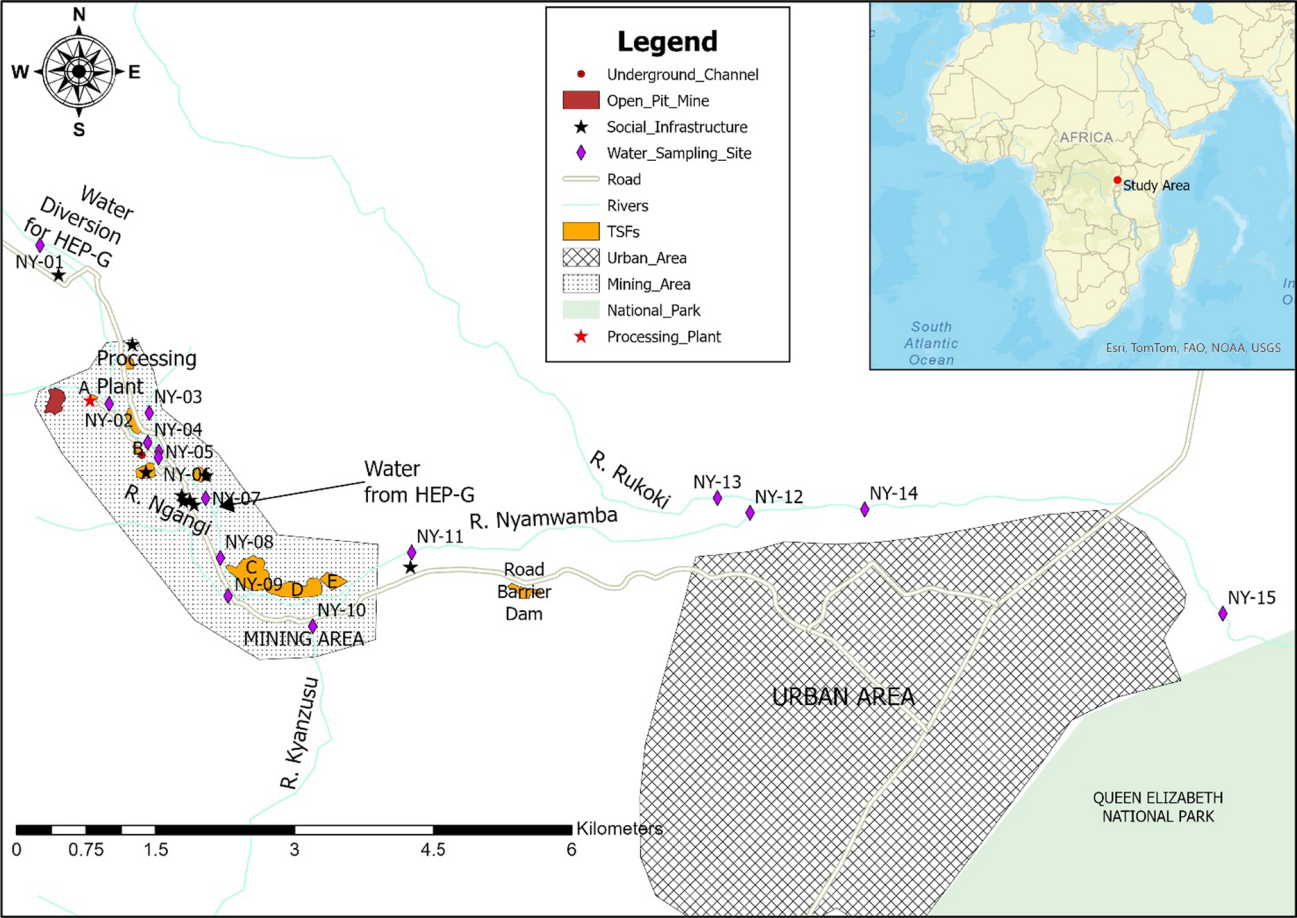
Location of the study area showing mining and urban areas, and water sampling sites (TSFs – Tailing Storage Facilities; HEP-G – Hydroelectric Power Generation)

The community within the River Nyamwamba catchment area use the river water for drinking, bathing, swimming, domestic and irrigation purposes (Kwetegyeka et al., 2014). However, the river is affected by copper mining that took place from 1956 to 1982 at the Kilembe Mine. The mining left more than 3.72 million cubic meters of tailings deposited in several tailings storage facilities (TSF) in the floodplains of River Nyamwamba (Abraham & Susan, 2017) (Fig. 1). Erosion of these tailings and discharge of underground mining-affected water, together with agricultural runoff and untreated domestic wastes, can pose potential threats to water quality and food crops grown in the vicinity (Zhang et al., 2023). Most residents in this densely populated area are subsistence farmers who rely on agriculture as their primary source of livelihood (Abraham & Susan, 2017). Most food crops, vegetables, and fruit trees, including bananas, maize, cassava, yams, potatoes, Amaranthus species, tomatoes, onions, beans, avocados, and mangoes grown downstream of the mining area could potentially be affected (Mwesigye et al., 2016).

The River Nyamwamba drains into Lake George, lying on the Equator within the Queen Elizabeth National Park, with a rich fish diversity of over 57 fish species supporting commercial fishery (Lwanga et al., 2003). The lake boasts a rich, biodiverse wetland on the northern part of the lake shore, designated by the Government of Uganda in 1988 as a Ramsar site (Denny et al., 1995; Lwanga et al., 2003). The lake is alkaline and extremely eutrophic with high evaporation rates (Owor et al., 2007). The largest flow into Lake George comes from the River Nyamwamba (Hartwig et al., 2005).

### Water sampling and determination of physicochemical water parameters

Field sampling was carried out in the dry season in January 2024. The water sample collection was conducted solely in the dry season because it provided a safe and accessible sampling environment. The study area is bushy and prone to flooding during wet seasons, making access to some locations within the study area impossible. Coupled with a time limitation on the number of times trips could be made to the study area, the dry season was preferred over the wet season for sample collection to avoid being stuck in the mud and encountering dangerous water levels. The sampling spanned two weeks in the field.

Nineteen sampling sites were established: nine along the River Nyamwamba, six on its tributaries, and four in Lake George. The sampling sites' location coordinates and altitudes were recorded in the field with a GARMIN GPSMAP 66sr MULTI-BAND Global Positioning System Instrument (Table S1). Water samples were collected from the River Nyamwamba upstream of the mine and downstream just before the river flowed into the Queen Elizabeth National Park on its way to Lake George, before and after tributary confluences, from tributaries just before joining the River Nyamwamba (Fig. 1), and from Lake George, randomly.

Water samples were collected in 30 mL high-density polyethylene bottles. Duplicates and field-blind samples were taken after every 5 sampling sites. Water was collected into a bucket, drawn with a clean syringe, and passed through a 0.45 µm filter into the 30 mL bottle. The water samples were acidified with 2 mL of 5 M HNO_3_ at the respective sampling sites. For field-blind samples, the bottles were filled with deionised water to the brim and airtightly capped from the laboratory and left overnight. In the field, the field-blind water bottle was exposed for the duration of sampling at a site where the field-blind sample was collected. The field-blind samples were also acidified with 2 mL of 5 M HNO_3_ at the respective sampling sites before being capped.

At each sampling site, pH was measured in triplicate using a calibrated HI 98194 multiparameter meter. Calibration was checked after every five sites with certified reference material, and the equipment was rinsed with deionised water after each use. Accuracy and precision for these measurements ranged from 0–3% and 0–5%, respectively.

### Laboratory analysis

The concentrations of PEMs (Al, As, Cd, Co, Cr, Cu, Fe, Mn, Mo, Ni, Pb, and Zn) were measured using an Agilent 7700 × ICP-MS in helium mode to reduce interferences. Calibration was performed using certified multielement standards, with additional quality control through reference materials, duplicates, blanks, and blind field samples. A Rhodium internal standard was used to correct for instrumental drift and matrix effects. Nearly half the blank and blind samples were undetectable; the rest showed minimal contamination, likely due to airborne particles. Method accuracy ranged from 1–9% and precision from 0–5%.

All the blank values and 48% of the field blind samples were undetectable. Other blind field samples (52%) showed very low concentrations compared to the respective actual water samples, suggesting contamination during sampling, possibly from airborne contamination, as visual particles were evident at the surface of some blind samples before capping at the sites.

### Quality assurance

For quality control, at each sampling site, the HI 98194 multiparameter meter was rinsed with deionised water after use, and the calibration was checked after every five sites with certified reference material. Before sampling, a plastic bucket was rinsed three times with the river water to be sampled. The water bottle was also rinsed three times, however, with filtered water for sampling. For field-blind samples, the water bottles were rinsed three times with deionised water in the laboratory. The water samples in 30 mL high-density polyethylene bottles were placed in well-labelled polyethylene sample bags, packed in a paper box, and carried to the laboratory at the Environment and Sustainability Institute, University of Exeter, UK, then stored at 4 °C until analysis.

The Agilent 7700 × ICP-MS was calibrated with a multielement calibration solution from ESSLAB (certified reference material) containing 1.6 µg/L, 8 µg/L, 40 µg/L, 200 µg/L, and 1000 µg/L of the analysed PEMs. The 40 µg/L certified reference material, duplicate samples, field blind samples, and procedural blanks were analysed to assess the method's accuracy and precision. The certified reference material and a procedural blank were determined between each batch of 10 water samples. In addition, a 500 µg/L solution of Rh (Rhodium) was run in parallel to provide an internal standard used for correcting any instrumental drift and matrix effects that could enhance or decrease the signal.

### Water quality assessment

#### Heavy metal evaluation index (HEI)

Heavy metal evaluation index estimates the intensity of PEMs in water samples by evaluating the overall contamination level, considering the concentrations of various PEMs against their respective maximum allowable concentration values (regulatory compliance). The HEI value is calculated by summing up the ratios of the measured concentrations to the maximum permissible concentration for each PEM (Ahirvar et al., 2023; Badeenezhad et al., 2023). The equation is as follows:

1 $$HEI = \mathop \sum \limits_{i = 1}^{n} \frac{{C_{i} }}{{MPL_{i} }}$$

where C_i_ is the measured concentration of the i^th^ PEM, MPL_i_ is the Maximum Permissible Limit for the i^th^ PEM, and n is the number of PEMs.

A value of HEI ≤ 40 denotes a low level of PEM contamination, 40 < HEI ≤ 80 indicates a medium level of contamination, and HEI > 80 signifies a high level of PEM contamination posing a significant risk to water quality and a potential threat to human health (Badeenezhad et al., 2023).

#### Pollution Load Index (PLI)

Pollution load index is a tool for assessing the overall contamination level in a particular area by calculating the geometric mean of contamination factors (CFs) for various contaminants (Jiang et al., 2020; Ma et al., 2023). The PLI is the n^th^ root of the product of the contamination factor (CF) for each analysed PEM, where n is the total number of analysed PEMs. This study used the PLI to ascertain the environmental risk of the River Nyamwamba water. The equation below was used to calculate PLI.

2 $$PLI = \sqrt[n]{{C_{f1} \times C_{f2} \times C_{f3} \times \cdots \times C_{fn} }}$$

3 $$C_{fi} = \frac{{C_{mi} }}{{C_{bi} }}$$

where C_f_ is the contamination factor of the i^th^ PEM, n is the number of PEMs, C_mi_ is the measured concentration of the i^th^ PEM, and C_bi_ is the background concentration of the i^th^ PEM, which was taken to be the average concentration value at sampling sites NY-01 and NY-13 that were considered uncontaminated. A value of PLI < 1 denotes no contamination, 1 ≤ PLI < 2 indicates moderate contamination, 2 ≤ PLI < 5 shows severe contamination, and PLI ≥ 5 signifies extreme contamination (Jiang et al., 2020).

#### Water quality index (WQI)

Water quality index is a tool that comprehensively assesses the water quality of a river (Meng et al., 2016; Sahu & Sikdar, 2007; Wang et al., 2017; Xiao et al., 2019). The index rates the combined influence of different water quality parameters and, thus, is used to assess the suitability of water for human consumption (Sahu & Sikdar, 2007). The computation of WQI involves assigning a weighting to each analysed PEM ranging from 1 to 5, based on its relative toxicity in the overall quality of water for drinking purposes, where 5 denotes highly toxic and 1, less toxic (Khadija et al., 2021; Sahu & Sikdar, 2007). The WQI was used to assess the River Nyamwamba water quality for drinking purposes because Uganda does not have its own water quality index. The calculation utilises the equation below.

4 $$WQI = \mathop \sum \limits_{i = 1}^{n} \left( {\frac{{C_{i} \times W_{i} }}{{S_{i} \times \sum W_{i} }} \times 100} \right)$$

where C_i_ is the concentration of the i^th^ parameter in the water sample, W_i_ is the weightage assigned to the i^th^ parameter (pH = 4, Al = 2, As = 5, Co = 2, Cu = 2, Fe = 4, Mn = 5, Mo = 3, Ni = 4, Pb = 5, and Zn = 1) (Meng et al., 2016), ΣW_i_ is the sum of all parameter weights (36), and S_i_ represents the maximum permissible values for the i^th^ parameter set by the Uganda National Bureau of Standards. The values of WQI are classified as excellent water quality when WQI < 50, good water quality when 50 ≤ WQI < 100, poor water quality when 100 ≤ WQI < 200, very poor water quality when 200 ≤ WQI < 300, and water unsuitable for drinking when WQI ≥ 300 (Khadija et al., 2021; Sahu & Sikdar, 2007).

#### Canadian council of ministers of the environment water quality index (CCME WQI)

The CCME WQI is a modification of the British Columbia water quality index model (BC WQI). It reduces the multivariate nature of water quality data by mathematically combining all water quality measures, including the scope, frequency, and amplitude of excursions from objectives, and producing a single value for water quality that is easy and simple to understand (CCME, 2001). The CCME WQI has been utilised for various surface water bodies and is the most common and widely used water quality index because of the flexibility in choosing the water quality parameters to be included in the model (Uddin et al., 2021). The CCME WQI model requires a minimum of four water quality parameters sampled at least four times to calculate index values. Nevertheless, the non-inclusion of a sub-index calculation component and parameter weightings is considered a major model deficiency (Uddin et al., 2021). The CCME WQI is calculated from using Eqs. 5 to 11 (CCME, 2001):

5 $$CCME WQI =100 - \left(\frac{\sqrt{\left({F}_{1}^{2}+ {F}_{2}^{2}+{F}_{3}^{2}\right)}}{1.732}\right)$$

F_1_ is the scope representing the percentage of parameters whose objectives are not met.

6 $$F_{1} = \left( {\frac{{Number\;of\;failed\;parameter}}{{Total\;number\;of\;parameters}}} \right) \times 100$$

F_2_ is the frequency, which represents the percentage of the frequency with which the objectives are not met.

7 $$F_{2} = \left( {\frac{{Number\;of\;failed\;tests}}{{Total\;number\;of\;tests}}} \right) \times 100$$

F_3_ is the amplitude representing the amount by which the objectives are not met and is calculated by an asymptotic function below.

8 $$F_{3} = \left( {\frac{nse}{{nse + 1}}} \right) \times 100$$

where nse is the normalised sum of excursions calculated as.

9 $$nse = \frac{{\sum\nolimits_{{i = 1}}^{n} e xcursion_{i} }}{{Total\;number\;of\;tests}}$$

When the test value must not exceed the objective:

10 $$excursion_{i} = \left( {\frac{{Failed\;Test\;Value_{i} }}{{Objective_{j} }}} \right) - 1$$

For the cases in which the test value must not fall below the objective:

11 $$excursion_{i} = \left( {\frac{{Objective_{j} }}{{Failed\;Test\;Value_{i} }}} \right) - 1$$

The CCME WQI classifies water quality as excellent when CCME WQI > 95, good when 80 ≤ CCME WQI < 95, fair when 65 ≤ CCME WQI < 80, marginal when 45 ≤ CCME WQI < 65, and poor when CCME WQI < 45.

### Environmental risk assessment

Environmental risks were estimated using the Ecological Risk (Er) and the Potential Ecological Risk Index (PERI). These are useful in evaluating PEM toxicity and biomagnification potential (Ahirvar et al., 2023). This study used PERI to estimate the potential environmental risks of PEMs in the River Nyamwamba water. The calculation follows Eqs. 12 and 13 (Ahirvar et al., 2023; Hakanson, 1980).

12 $$PERI = \mathop \sum \limits_{i = 1}^{n} Er_{i}$$

13 $$Er_{i} = T_{ri} \times C_{fi}$$

where Er_i_ is the ecological risk for the i^th^ PEM, T_ri_ is the toxic‐response factor for the i^th^ PEM;- T_r_ values for Al = 5, As = 10, Cd = 30, Co = 5, Cr = 2, Cu = 5, Fe = 1, Mn = 1, Mo = 15, Ni = 5, Pb = 5, and Zn = 1 (Hakanson, 1980; Zhang & Liu, 2014), C_fi_ is the contamination factor of the i^th^ PEM as calculated in Eq. 3 above, however, replacing concentration of i^th^ PEM with mean concentration of the i^th^ PEM from at least five sampling points (Hakanson, 1980).

The value of Er_i_ < 40, PERI < 150, indicates low potential ecological risk; 40 ≤ Er_i_ < 80, 150 < PERI < 300 indicate moderate potential ecological risk; 80 ≤ Er_i_ < 160, 300 ≤ PERI < 600 shows considerable potential ecological risk; 160 ≤ Er_i_ < 320, 600 ≤ PERI < 1200 exhibit high potential ecological risk; and Er_i_ ≥ 320, PERI ≥ 1200 reveal extremely high potential ecological risk (Jiang et al., 2020; Ma et al., 2023).

#### Human health risk assessment

Contaminants, ecosystems, and human health relationships in the environment can be assessed by estimating the human health risk (Panda et al., 2023). Human health risk assessment is helpful in the prevention and management of hazards. It involves hazard identification, exposure assessment, toxicity assessment, and risk characterisation (Kamunda et al., 2016; WHO, 2021) of the populace exposed to unhealthy or hazardous substances in contaminated environmental media (Oni et al., 2022; Sharma, 2020). Exposure is the amount of a contaminant absorbed by the human body through various pathways (Cocârţă et al., 2016). The non-carcinogenic and carcinogenic health risks were calculated per US EPA guidelines using the PEM concentrations, reference dose values, the oral cancer slope factors for respective PEMs through the ingestion pathway, and the absorbed cancer slope factors for respective PEMs through the dermal contact pathway (US EPA, 2004; Badeenezhad et al., 2023).

#### Chronic daily dose (CDD)

Exposure assessments were conducted by estimating the average chronic daily doses/intakes of PEMs through ingestion and dermal contact with the River Nyamwamba water for adults and children within the study area. The Chronic Daily Dose (CDD) (mg/kg-day) via ingestion and the Dermally Absorbed Dose (DAD) (mg/kg-day) via dermal contact were calculated using the following governing equations per US EPA ( HYPERLINK "sps:refid::bib69|bib70" ):

The Chronic Daily Dose (CDD):

14 $$CDD = \frac{C \times IR \times ED \times EF}{{AT \times BW}}$$

The Dermally Absorbed Dose (DAD):

15 $$DAD = \frac{{C \times K_{p} \times ED \times EF \times ET \times SA}}{AT \times BW}$$

where C is the concentration of the ith PEM (mg/L), IR is the ingestion rate (L/day), ED is the exposure duration (years), EF is the exposure frequency (days/year), AT is the average time (days), BW is the body weight (kg), Kp is the dermal permeability coefficient of the i^th^ PEM, ET is the exposure time per event (hr), and SA is the exposed skin area (cm^2^).

#### Non-cancer risk

Non-carcinogenic hazards were characterised with the Hazard Quotient (HQ) and Hazard Index (HI). Hazard quotient is a unitless number stating the probability of an individual experiencing adverse health effects (Kamunda et al., 2016), which is the ratio of the average chronic daily dose (CDD) (mg/kg-day) to the chronic reference dose (RfD) (mg/kg-day) of a specific PEM for a single pathway as per risk guidelines of the US EPA ().

16 $$HI = \sum HQ$$

17 $$HQ = \left( {HQ_{ing} + HQ_{dermal} } \right)$$

18 $$HQ_{ing} = \frac{CDD}{{RfD_{ing} }}$$

19 $$HQ_{dermal} = \frac{DAD}{{RfD_{ABS} }}$$

20 $$RfD_{ABS} = RfD_{ing} \times ABS_{g}$$

where HQ_ing_ and HQ_dermal_ are Hazard Quotients for PEM uptake through ingestion and dermal contact, respectively, RfD_ing_ is the Reference Dose for ingestion, RfD_ABS_ is the Absorbed Reference Dose for dermal contact, and ABS_g_ is the fraction of contaminant absorbed in the gastrointestinal tract.

Adequate preventive and protective measures must be undertaken for individual values of HQ ≥ 1, indicating a potential risk to human health (Kicinska and Wikar, 2023). The risk factors are negligible for HQ < 1, moderately significant for 1 = HQ < 10, and highly significant for HQ ≥ 10.

The Hazard Index (HI) is the summation of all the HQs for individual PEM (Wang et al., 2017). No harmful effects exist when HI < 1; otherwise, there is a potentially harmful impact on human health (Badeenezhad et al., 2023; Joodavi et al., 2021; Wang et al., 2017; Xiao et al., 2019).

#### Cancer risk (CR)

The carcinogenic health risk (CR) is the probability of an individual developing cancer during their lifetime through exposure to the potential carcinogen (Kamunda et al., 2016; Kim et al., 2019; Oni et al., 2022). The CR was estimated using the oral and absorbed cancer slope factors via ingestion and dermal contact pathways, respectively (US EPA, 2004; Badeenezhad et al., 2023). The cancer slope factors for Al, Co, Cu, Fe, Mn, Mo, and Zn could not be found in literature, and the aqueous Cd and Cr concentrations were below their respective detection limits of 0.29 µg/L and 0.05 µg/L, respectively. Therefore, Al, Cd, Co, Cr, Cu, Fe, Mn, Mo, and Zn could not be considered for carcinogenic health risk assessments.

21 $$CR = \left( {ELCR_{ing} + ELCR_{dermal} } \right)$$

22 $$ELCR_{ing} = CDD \times CSF_{oral}$$

23 $$ELCR_{dermal} = DAD \times CSF_{ABS}$$

24 $$CSF_{ABS} = \frac{{CSF_{oral} }}{{ABS_{g} }}$$

where CR is the cancer risk, ELCR_ing_ and ELCR_dermal_ are the excess lifetime cancer risks through ingestion and dermal contact, respectively, CDD is the chronic daily dose for ingestion (mg/kg-day), DAD is the dermally absorbed dose for dermal contact (mg/kg-day), CSF_oral_ and CSF_ABS_ are the oral and absorbed cancer slope factors (risk per mg/m^3^), respectively, and ABS_g_ is the fraction of contaminant absorbed in the gastrointestinal tract (dimensionless).

The carcinogenic risk is unacceptable when CR > 1 × 10^–4^, negligible when CR < 1 × 10^–6^, and otherwise, acceptable (Table S3) (US EPA, 2004; Dashtizadeh et al., 2019; Kicinska and Wikar, 2023; US EPA, 2023).

### Statistical analysis

Data analyses and statistical tests were performed using Microsoft Excel (Microsoft® Excel® for Microsoft 365 MSO (Version 2408 Build 16.0.17928.20114) 64-bit) and the IBM Statistical Package for the Social Sciences (SPSS) version 29 for Windows. Descriptive statistics were used to interpret the obtained data. Factor analysis/principal component analysis (FA/PCA), as well as the hierarchical agglomerative cluster analysis (CA) employing the average linkage method (between groups), with the original values of variables standardised using z-score transformation, were used to explore the possible relationships and sources of PEMs in the River Nyamwamba. The suitability of the data for factor analysis was tested by Kaiser–Meyer–Olkin (KMO) and Bartlett's sphericity test, where KMO > 0.5 and *P* < 0.001 is generally considered acceptable (Wang et al., 2017).

## Results and discussion

### Water quality assessment

#### Physicochemical characteristics

**Fig. 2:**
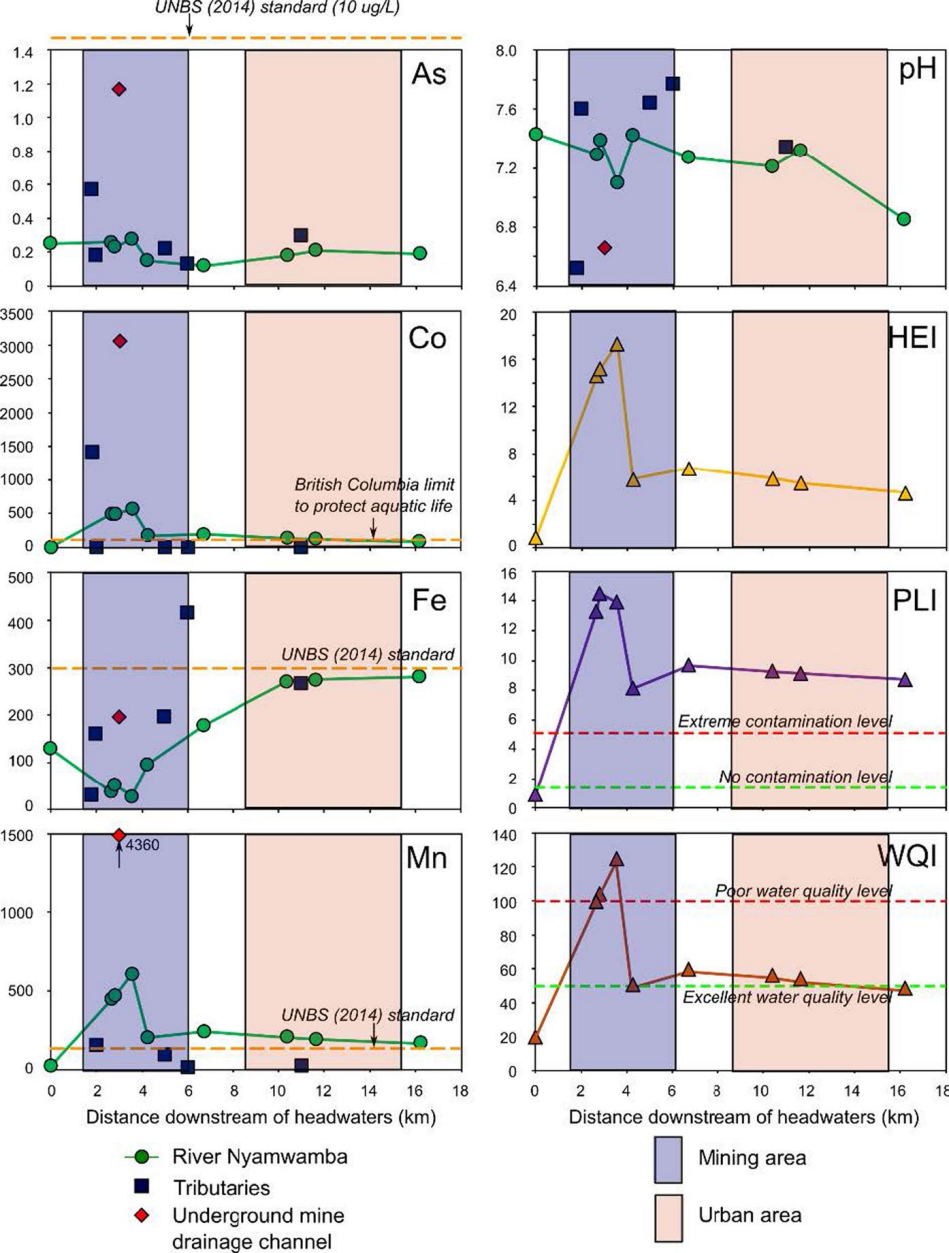
Concentrations of filtered As, Co, Fe, and Mn (in µg/L), aqueous pH, and HEI, PLI, and WQI indices with distance downstream of the sampling site NY-01 (upstream of the mining area along the River Nyamwamba). The British Columbia limit to protect aquatic life is 110 ug/L, taken from Nagpal (2004)

**Fig. 3:**
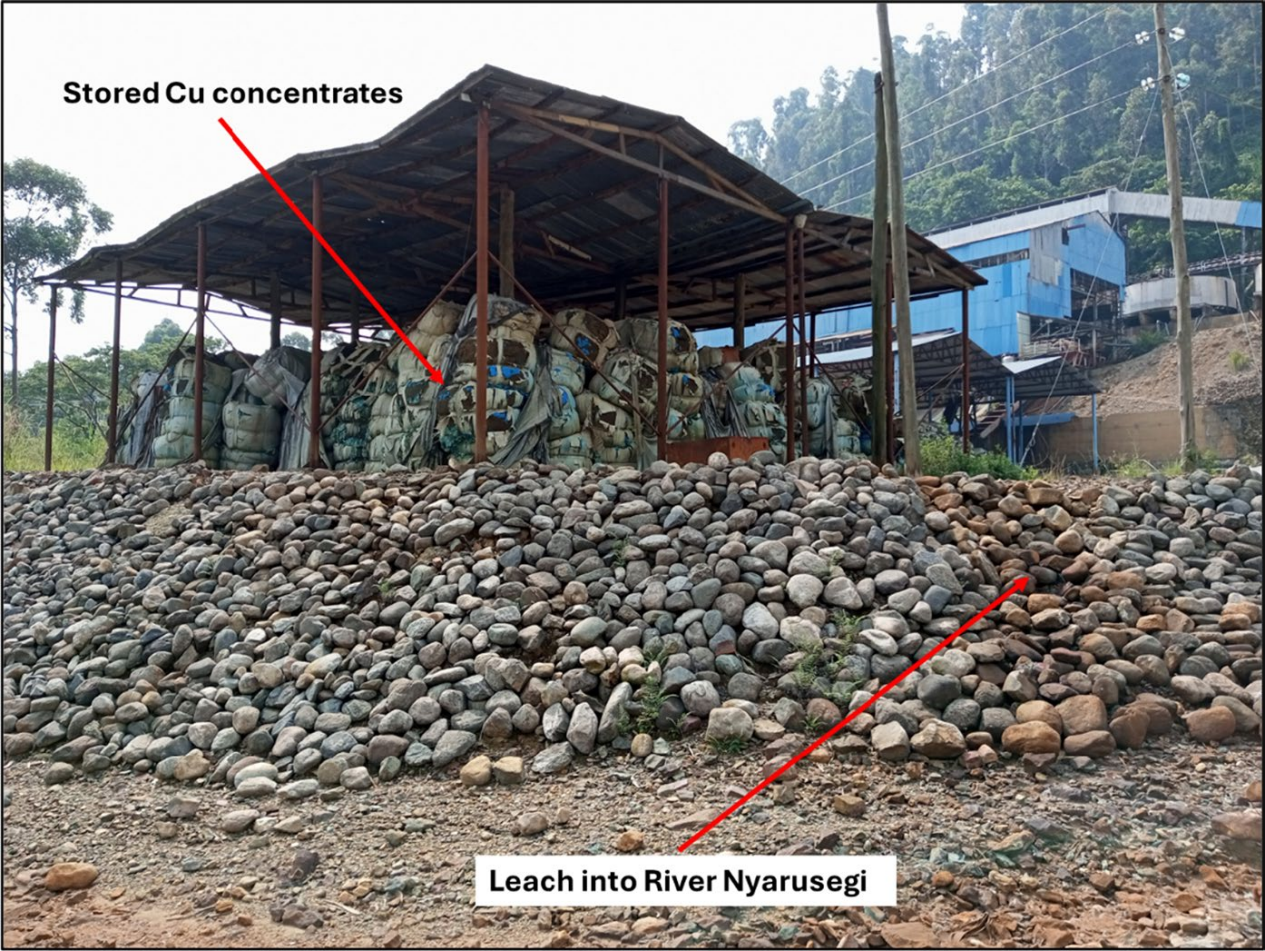
Photograph showing over 800 tonnes of Cu concentrates stored at a former processing plant that leach into the River Nyarusegi

Filtered concentrations of selected PEMs and pH for the 19 water samples collected from the Nyamwamba catchment are shown in Table 1 and plotted with distance downstream and possible source inputs in Fig. 2. The plots for Mn and Ni show a similar trend to that of Co (Table 1). The highest pH value (7.77) for the rivers was recorded at sampling site NY-10 on the River Kyanzusu tributary, probably due to non-contamination with PEMs at this site. Whereas, the lowest (6.85) was at sampling site NY-15 downstream of the Kilembe Mining licence. All aqueous pH values for the river waters were within the acceptable limits of 5.5–9.5 set by the Uganda National Bureau of Standards (UNBS) for potable water (UNBS, 2014) and 6.5–8.5 set by the World Health Organisation (WHO) for drinking water (WHO, 2017) (Table 1). The decrease in pH of the River Nyamwamba downstream (Fig. 2) could be attributed to discharge from the underground mine drainage channel, leaching from TSF C, D, and E (Abraham & Susan, 2017; MWE, 2022), located within the floodplain of the river (Fig. 1), tributaries such as the River Nyarusegi, which traverses close to the TSF A and a former ore processing plant storing over 800 tonnes of Cu concentrates (Fig. 1, 3), and the River Ngangi, which ferries domestic wastes from Kilembe town. Some of the water from the River Nyamwamba was diverted upstream about 50 m below the sampling site (NY-01) for hydroelectric power generation (HEP-G) and redirected back to the river downstream between sampling sites NY-07 and NY-08 (Fig. 1). This could be the reason for the increase in pH value from 7.1 to 7.4 (Fig. 2). The sampling site NY-15 presented the lowest pH probably due to the influence of the mining and the associated tailings together with various activities such as sand and stone (pebble) mining, as well as the washing of cars, motorcycles, bicycles, and clothes in the river (Abraham & Susan, 2017; Masereka et al., 2022; MWE, 2022), which can supply PEMs from diesel leaks and detergents (Aonghusa & Gray, 2002). The obtained pH values were comparable to 7.30 (Hartwig et al., 2005) and 6.82–7.07 (Kwetegyeka et al., 2014), which were previously reported for the River Nyamwamba. In contrast, all the pH values obtained for Lake George water in this study exceeded the maximum limit of 8.5 set by the WHO for drinking water (WHO, 2017), yet were within the acceptable limit of 9.5 set by the UNBS for potable water (UNBS, 2014). The obtained pH values in Lake George water increased towards the lake centre and were comparable to the values 9.9, 8.8, and 7.1–9.9 reported previously by Denny et al. (1995), Owor et al. (2007) and Holmberg et al. (2011), respectively.

**Table 1:** Filtered (< 0.45 µm) concentrations of potentially ecotoxic metal(loid)s (PEM) in the River Nyamwamba, its tributaries, an underground mine drainage channel and Lake George

	Sample Number	pH	Al (µg/L)	As (µg/L)	Co (µg/L)	Cu (µg/L)	Fe (µg/L)	Mn (µg/L)	Mo (µg/L)	Ni (µg/L)	Pb (µg/L)	Zn (µg/L)
*River Nyamwamba*												
	NY-01	7.43	33.0	0.25	< 0.053	1.91	130	17.5	0.55	< 0.098	0.41	< 1.46
	NY-04	7.29	27.8	0.26	**494**	181	38.2	**444**	0.41	**104**	0.15	49.8
	NY-05	7.39	39.3	0.23	**493**	159	51.8	**467**	0.46	**109**	0.19	51.6
	NY-07	7.10	37.0	0.28	**564**	150	28.1	**604**	0.56	**113**	0.11	51.4
	NY-08	7.42	40.4	0.15	**171**	101	96.7	**200**	0.41	**32.6**	0.07	14.2
	NY-11	7.27	62.0	0.12	**193**	77.7	179	**240**	0.42	**31.4**	0.14	17.1
	NY-12	7.21	84.8	0.18	**137**	78.9	272	**205**	0.40	**22.8**	0.13	8.58
	NY-14	7.32	84.7	0.21	**123**	75.2	275	**190**	0.41	**20.2**	0.17	6.86
	NY-15	6.85	90.8	0.19	84.3	66.1	282	**165**	0.40	14.8	0.17	11.3
*Tributaries and Other Outputs*												
Nyarusegi	NY-02	6.52	21.8	0.57	**1420**	1170	31.9	**1130**	0.54	**326**	0.42	216
Kanyaruboga	NY-03	7.60	16.4	0.18	3.26	1.44	161	**148**	0.82	0.88	0.22	< 1.46
UMDC	NY-06	6.66	25.4	1.17	**3060**	223	198	**4360**	2.56	**514**	0.08	182
Ngangi	NY-09	7.54	72.2	0.22	< 0.053	0.12	197	88.1	0.41	< 0.098	0.25	3.03
Kyanzusu	NY-10	7.77	33.6	0.13	< 0.053	0.12	**415**	7.59	0.36	< 0.098	0.17	2.64
Rukoki	NY-13	7.34	61.0	0.30	< 0.053	< 0.453	267	22.1	0.32	< 0.098	0.13	4.18
*Lake George*	LG-01	9.01	15.16	0.17	< 0.053	< 0.453	25.56	2.73	< 0.238	< 0.098	0.16	12.94
	LG-02	9.26	10.19	0.21	< 0.053	< 0.453	13.35	1.56	< 0.238	< 0.098	0.21	1.83
	LG-03	8.92	24.95	0.16	< 0.053	< 0.453	35.32	1.91	< 0.238	< 0.098	0.71	3.49
	LG-04	8.91	9.72	0.24	< 0.053	< 0.453	12.81	0.68	< 0.238	< 0.098	0.20	3.53
*Water Quality Standards*												
UNBS, 2014			200	10	110*	1000	300	100	70	20	10	5000
WHO, 2017			200	10	NR	2000	300	100	70	70	10	3000

UMDC: Underground mine drainage channel. Concentrations in bold exceed UNBS and WHO water quality standards. * from Nagpal (2004)

**Table 2:** Loading for Factors 1 and 2 from Principal Component Analysis

PEM	Factor 1	Factor 2
Mn	0.996	
Co	0.972	
As	0.962	
Mo	0.947	
Ni	0.929	0.349
Zn	0.760	0.600
Cu		0.834
Pb		0.730
Fe		-0.713
Al	-0.339	-0.563
Variance	60.5%	20.4%
Cumulative	60.5%	80.9%

Extraction method: Principal component analysis. Rotation method: Varimax with Kaiser normalisation

#### Aqueous PEM concentrations

Cobalt, Mn, and Ni had the highest concentrations of all the PEMs, and Cd and Cr were undetected in all water samples. The concentrations of Al, As, Cu, Fe, Mo, Pb, and Zn in the River Nyamwamba water were within permissible limits for potable water set by the UNBS (UNBS, 2014) and for drinking water set by the WHO (WHO, 2017). However, the Fe concentration of the River Kyanzusu tributary (416 µg/L) was above the permissible limit of 300 µg/L set by both the UNBS and the WHO. The reason for this elevated concentration was unknown. Similarly, the filtered Cu concentration (1,170 µg/L) of the River Nyarusegi tributary exceeded the permissible limit of 1,000 µg/L set by the UNBS for potable water, yet was within the acceptable limit (2,000 µg/L) of the WHO for drinking water. This high concentration may be due to the leaching of copper concentrate at the former processing plant (Fig. 3) and TSF A into the River Nyarusegi (Abraham & Susan, 2017).

Seventy-three percent (%) of filtered aqueous Mn concentrations of the River Nyamwamba samples exceeded the permissible limit of 100 µg/L set by both the UNBS (UNBS, 2014) and the WHO (WHO, 2017). The highest Mn concentrations were recorded at NY-06 (4,360 µg/L), located on the underground mine drainage channel, followed by NY-02 (1,130 µg/L), located along the River Nyarusegi that traverses close to the TSF A and a former ore processing plant. The NY-01 sampling site, located upstream of the mining area, was the only site whose Mn concentration (17.5 µg/L) was within acceptable limits for both UNBS and WHO. In comparison, water samples from Lake George had a very low mean Mn concentration (1.72 µg/L). The mean aqueous River Nyamwamba Mn concentration (281 µg/L) was higher than the 60 µg/L and 100 µg/L reported for River Benin in Nigeria (Ezemonye et al., 2019) and River Musonoie in DRC (Atibu et al., 2013), respectively, within a similar tropical climate as this study area, and 135 µg/L reported for River Kafue in Zambia within the Copperbelt (Kribek et al., 2023). However, the mean aqueous River Nyamwamba Mn concentration was lower than the 550 µg/L reported for the River Chari (Nambatingar et al., 2017) in Chad, with a similar tropical climate, and 285 µg/L reported for the River Tigris in Turkey (Varol and Sen, 2012), also flowing close to a copper mine.

Sixty percent (%) of the River Nyamwamba water samples exhibited Ni concentrations that exceeded the permissible limit of 20 µg/L for potable water by the UNBS (UNBS, 2014), though only 33.3% were above the acceptable limit for drinking water (70 µg/L) set by the WHO (WHO, 2017). The highest Ni concentrations were recorded at NY-06 (514 µg/L), located on the underground mine drainage channel, followed by NY-02 (326 µg/L), located along the River Nyarusegi that traverses close to the TSF A and a former ore processing plant. The sampling sites with Ni concentrations within acceptable limits were NY-01 (undetected) and NY-15 (14.8 µg/L) upstream and downstream, respectively, of the mining area. Filtered Ni concentration decreased to within the permissible limit of the UNBS at the sampling site NY-15 due to dilution from the waters from the tributaries and HEP-G. In contrast, Ni was not detected in Lake George. The mean filtered Ni concentration (48.8 µg/L) in the River Nyamwamba water in this study was higher than the 45 µg/L reported for the River Tigris in Turkey (Varol and Sen, 2012), which also flowed near a copper mine. However, the mean filtered Ni concentration was lower than the 650 µg/L and 420 µg/L reported for the tropical Rivers Chari in Chad (Nambatingar et al., 2017) and Benin in Nigeria (Ezemonye et al., 2019).

No guideline value for acceptable limits for Co in water was readily accessible. Therefore, a 110 µg/L recommended guideline for British Columbia to protect aquatic life from the acute toxic effects of Co (Nagpal, 2004) was used to assess the Co concentration in this study. Sixty percent (%) of the River Nyamwamba water samples had filtered Co concentrations that exceeded the 110 µg/L limit. The highest Co concentrations were recorded at NY-06 (3,060 µg/L), located on the underground mine drainage channel, followed by NY-02 (1,420 µg/L), located along the River Nyarusegi that traverses close to the TSF A and a former ore processing plant. Like Ni, the only sites that exhibited Co concentrations within acceptable limits were NY-01 (undetected) and NY-15 (84.3 µg/L) upstream and downstream, respectively, of the mining area. In comparison, Co was not detected in Lake George. The mean concentration of Co (251 µg/L) in the River Nyamwamba water was higher than the 40 and 200 µg/L concentrations reported for the mining-affected River Tigris in Turkey (Varol and Sen, 2012) and the River Musonoie in DRC (Atibu et al., 2013), respectively.

Overall, concentrations of PEMs in the upstream site (NY-01) were lower than those within the mining area. The elevated Co, Mn, and Ni concentrations could be attributed to leachate from the processing plant and the discharge from the underground mine drainage channel (Abraham & Susan, 2017; MWE, 2022). These elevated concentrations made the river water unsuitable for domestic and drinking purposes, suggesting that remediation was required, especially in the River Nyarusegi and for the discharge from the underground mine drainage channel. By contrast, Co and Ni were undetected in Lake George water, and the mean concentration of Mn in the lake water was over one hundred sixty-three times lower than that of River Nyamwamba water. This could be attributed to (1) biological activity in the wetland on the lake shores (Denny et al., 1995), which the River Nyamwamba flows through before entering Lake George, (2) the alkaline (mean pH 9.02) and eutrophic nature of Lake George, coupled with the high temperatures promoting PEM precipitation and removal from the water, and 3) dilution from the waters from other inflows (such as rivers Mubuku, Mpanga, Ruimi, Hima, Nsonge, and Dura) into the lake.

#### Sources of PEMs to the River Nyamwamba

Factor analysis (FA) /principal component analysis (PCA) and cluster analysis (CA) were undertaken to derive additional information about the sources of the PEMs. The KMO and Bartlett test results were 0.594 (> 0.5) and 0.000 (< 0.001), respectively, indicating that the PEM sources can be analysed with PCA (Wang et al., 2017). Two principal components (factors) with eigenvalues exceeding one were extracted. Features with factor loadings greater than 0.5 were considered significant for interpreting each component. The FA/PCA results are presented in Table 2.

Factors 1 and 2 accounted for 60.5% and 20.4% of the variance, respectively, explaining 80.9% of the overall variance. The first principal component (Factor 1) was associated with strong positive loadings for Mn, Co, As, Mo, Ni, and Zn and a weak negative loading for Al. Factor loading is considered strong when > 0.75, and weak when < 0.50; otherwise, it is considered moderate (Wang et al., 2017). Factor 1 demonstrated significantly high variation coefficients, which indicated a similar origin. The elevated concentrations of As, Co, Mn, Mo, Ni, and Zn in the River Nyamwamba could be attributed to contamination by water from R. Nyarusegi (due to leachate from the processing plant and TSF A), discharge from the underground mine drainage channel and leachate inputs from TSF B, C, D, and E. Therefore, it is likely that the PEMs in Factor 1 were mainly derived from the Kilembe Copper Mine area. This conclusion aligns with earlier reports that leachate from the Kilembe Mine supplied PEMs to the River Nyamwamba (Abraham & Susan, 2017; Masereka et al., 2022; MWE, 2022).

The second principal component (Factor 2) was linked to a strong positive loading for Cu, moderate positive loadings for Zn and Pb, a weak positive loading for Ni, and moderate negative loadings for Al and Fe. The PEMs in Factor 2 exhibited lower variation coefficients than the PEMs in Factor 1, indicative of multiple origins. Therefore, the potential sources for PEMs in Factor 2 could be natural weathering, domestic waste disposals, sewage discharge, defecation in the riverbanks (Abraham & Susan, 2017; Kwetegyeka et al., 2014), and agricultural runoff from the unregulated and inappropriate use of pesticides by farmers cultivating on riverbanks (MWE, 2022), which have previously been proposed to be responsible for high PEM concentrations in the River Nyamwamba. Previous studies of the River Nyamwamba have also found Al, Cu, Pb, Fe, and Zn to come from natural sources (Abraham & Susan, 2017).

Cluster Analysis (CA) was used to identify related sampling sites and areas of contamination (Varol and Sen, 2012). The dendrogram in which the sampling sites were grouped into three statistically significant clusters at $\left({D}_{link}/{D}_{max}\right)\times 100<10$ is presented in Fig. S1. A smaller distance in the dendrogram indicates a closer relationship between the sites. Cluster 1 consisted of NY-01, NY-03, NY-04, NY-05, NY-07, NY-08, NY-09, NY-10, NY-11, NY-12, NY-13, NY-14, and NY-15 sampling sites, representing lowly contaminated sites. Cluster 2 contained the NY-02 sampling site, corresponding to a moderately contaminated site, and Cluster 3 contained the NY-06 sampling site, corresponding to a highly contaminated site. The Cluster 3 site was situated along the underground mine drainage channel. The Cluster 2 site was located along the River Nyarusegi (tributary) that flows near the processing plant and TSF A before joining the River Nyamwamba.

Similarly, CA was performed to detect the relationships among the PEMs and their possible sources (Varol and Sen, 2012). A dendogram was produced from the CA analysis (Fig. S2), where all the analysed PEMs were grouped into three statistically significant clusters at $\left({D}_{link}/{D}_{max}\right)\times 100<15$. A smaller distance in the dendrogram indicates a closer relationship between the PEMs. Cluster 1 included As, Co, Cu, Mn, Mo, Ni, and Zn as contaminants derived from a single source, probably the leachate from the processing plant and discharge from the underground mine drainage channel. Cluster 2 contained Pb possibly supplied by multiple sources, such as natural weathering, domestic waste disposals, sewage discharge, agricultural runoff, and riverbank defecation, but also influenced by the source in Cluster 1. Cluster 3 consisted of Al and Fe, possibly from natural and anthropogenic sources such as natural weathering, leachate from the mine, domestic waste disposals, and sewage discharge. Thus, the CA results are reasonably similar to the PCA in terms of source classification.

Overall, it is suggested that the spatial heterogeneity of anthropogenic activities (mining, agriculture, and domestic) has led to different characterisations of PEM contamination sources (Wang et al., 2017). Mining was responsible for As, Co, Cu, Mn, Mo, Ni, and Zn inputs to the River Nyamwamba system through discharges, leachate, and runoff waters, whereas agrochemicals and fertilisers used in agricultural activities within the riverbanks of River Nyamwamba likely supplied Mn, Fe, Co, Ni, Cu, Zn, As, and Pb through runoff and irrigation waters (Wang et al., 2017).

#### Water contamination assessment

The HEI, PLI, WQI, and CCME WQI results are presented in Table 3. HEI directly compares measured concentrations with maximum permissible limits defined by regulations. The potable water standard of the Uganda National Bureau of Standards (UNBS, 2014) was utilised as the maximum permitted limit for respective PEMs in water. The HEI results suggested that the River Nyamwamba water exhibited low levels of contamination, with all HEI values less than 40 (Table 3). The highest HEI value (17.3) in the River Nyamwamba water was presented at the NY-07 sampling site, and the lowest (0.9) was displayed at the NY-01 sampling site. Variations in HEI values were visible at the different sampling sites, especially at NY-04 (14.6), NY-05 (15.2), and NY-07 (17.3), which were 2.66 km, 2.82 km, and 3.56 km downstream of the sampling site NY-01, respectively. These sites experienced more pronounced contamination than the other sampling sites. Elevated HEI values could be attributed to the leachate from the processing plant and discharge from the underground mine drainage channel within Kilembe Mine.

The PLI results revealed that all sampling sites except NY-01 had values greater than 1 (Table 3), signifying extreme contamination. Sampling site NY-05 exhibited the highest PLI value of 14.5, demonstrating a more pronounced contamination load at this site, whereas the PLI value for the NY-01 site (0.9) signified moderate contamination upstream. The contrast between the HEI results (showing low contamination) and the PLI results (showing extreme contamination) is likely because HEI utilises maximum permissible limits for PEM concentrations (regulatory compliance), whereas PLI utilises baseline PEM concentrations (contamination with respect to background values). This therefore indicates a noticeable deviation from the baseline PEM concentrations in the river water, although not of concern for regulatory compliance.

The computed WQI values for the River Nyamwamba water ranged from 17.4 to 171 (Table 3), meaning that the samples can be classified into’excellent water quality’, ‘good water quality’, and ‘poor water quality’. Thirty-three % of the sampling sites (NY-04, NY-05, and NY-07), located within the mining area, exhibited poor water quality and are considered contaminated. Nevertheless, the upstream sampling site (NY-01) before the mining area was the only site considered uncontaminated. Moreover, the calculated CCME WQI value corresponding to the River Nyamwamba water was 73 (Table 3), corresponding to ‘fair’ water quality. Both the WQI and CCME WQI values obtained were comparable and indicate little concern over the water quality of River Nyamwamba in terms of regulatory compliance, just as the HEI predicted.

Overall, the multiple water contamination indices indicate that the water quality of River Nyamwamba worsened within the mining area, deviating from the baseline water quality, but not posing a threat at the time of sampling in terms of regulatory compliance.

### Environmental and human health risk assessments

#### Ecological Risk

The potential risks of Al, As, Co, Cu, Fe, Mn, Mo, Ni, Pb, and Zn were determined using the ecological risk (Er) and the Potential Ecological Risk Index (PERI). The results are presented in Table 4. Aluminium, As, Fe, Mn, Mo, Pb, and Zn had Er values less than 40 (Table 4), suggesting that they posed low potential ecological risks. However, Co, Cu, and Ni exhibited Er values above 320 (47,400, 463, and 5,080, respectively), indicating extremely high potential ecological risks. The PERI value obtained was 53,000, which exceeded 1,200 (Table 4), signifying extremely high potential ecological risks from PEMs.

**Table 3:** Results for water contamination indices

Sampling Sites	Distance Downstream (km)	HEI	PLI	WQI	CCME WQI
NY-01	0	0.85	0.91	17.4	72.9
NY-04	2.66	14.6	13.6	143.5	
NY-05	2.82	15.2	14.5	151	
NY-07	3.56	17.3	13.9	171	
NY-08	4.25	5.83	8.11	63.2	
NY-11	6.7	6.62	8.84	69.4	
NY-12	10.39	5.88	9.25	64.0	
NY-14	11.64	5.49	9.23	60.8	
NY-15	16.2	4.66	8.70	53.5

**Table 4:** Ecological risk (Er) and potential ecological risk index (PERI) for River Nyamwamba water

PEM	Al	As	Co	Cu	Fe	Mn	Mo	Ni	Pb	Zn
Mean concentration	55.5	0.21	251	98.9	150	281	0.45	49.8	0.17	23.5
Cb (Average of NY-01 & NY-13)	47.0	0.28	0.03	1.07	199	19.8	0.43	0.05	0.27	2.45
Cf	1.18	0.75	9,470	92.6	0.76	14.2	1.03	1,015	0.63	9.58
Tr	5.00	10.0	5.00	5.00	1.00	1.00	15.0	5.00	5.00	1.00
Er	5.91	7.46	47,400	463	0.76	14.2	15.4	5,080	3.17	9.58
**PERI** $(\sum {{\varvec{E}}}_{{\varvec{r}}})$	**53,000**									

#### Non-cancer risk

The Hazard Quotient (HQ) and Hazard Index (HI) were utilised to estimate the non-carcinogenic risks for children and adults (Table S4) based on RfD values as presented in Table S2. Non-carcinogenic risks to the populace are non-existent when HQ and HI values are less than 1, otherwise, potential non-carcinogenic effects exist (US EPA, 2004). The HQ values for Co, Cu, Fe, Mn, and Ni were greater than 1 in children and adults (Table S4), indicating potential non-carcinogenic risk in children and adults through oral and dermal contact with the River Nyamwamba water. The HQ values decreased in the order Co > Mn > Cu > Fe > Ni > Zn > Mo > As > Pb > Al. The HQ values for children were higher than the corresponding values for adults (Table S4). This could be attributed to the greater sensitivity of children to PEM exposure compared to adults, since children may absorb more PEMs from water during their outdoor play activities (Bello et al., 2019). The dermal contact with River Nyamwamba water presented higher HQ values than the oral pathway, signifying more pronounced non-carcinogenic risks with dermal contact compared to the ingestion pathway.

The cumulative risks due to the exposures to multiple PEMs through ingestion and dermal contact with the River Nyamwamba water were assessed by the Hazard Index (HI) to understand the overall potential for non-carcinogenic effects. The HI values obtained ranged from 8 to 1,149 for children and 1 to 167 for adults (Table S4). Cobalt, Cu, Fe, Mn, and Ni were responsible for HI values exceeding 1. Therefore, it can be concluded that Co, Cu, Fe, Mn, and Ni are the greatest contributors to chronic risks among the selected PEMs in the Kilembe area and the surrounding community. The HI values reported in this study for both children and adults surpassed the < 1 reported for Shanono and Bagwai gold mining areas in Nigeria (Bello et al., 2019).

#### Cancer risk

The human carcinogenic health risk/cancer risk (CR) due to exposure to As, Ni, and Pb was estimated using US Environmental Protection Agency (EPA) guidelines (US EPA 2004), and the results are presented in Table S5. The results suggested that the cancer risk from dermal contact with the river water was higher than the oral/ingestion pathway for both children and adults (Table S5). The cancer risks were more pronounced in children than in adults. The cancer risks from exposure to As, Ni, and Pb in both children and adults decreased in the following order: Ni > As > Pb. Lead presented the least cancer risk, with the highest contribution of 0.5% in both children and adults at the NY-01 sampling site (upstream of the mining area). Arsenic presented a 66.5% cancer risk contribution at the NY-01 sampling site. Nickel posed the greatest cancer risks, with nearly 100% contribution at all sampling sites in both children and adults, except at the NY-01 sampling site (33%).

The total carcinogenic/cancer risks ranged from 9.84 × 10^–5^ (corresponding to medium risk) to 2.24 × 10^–1^ (corresponding to extremely elevated risk) in children, and 1.44 × 10^–5^ (corresponding to low-medium risk) to 3.28 × 10^–2^ (corresponding to extremely elevated risk) in adults (Table S5 and Table S3). The River Nyawmamba water posed an extremely elevated cancer risk to both children and adults, except at the sampling site NY01 upstream of the mining area, where the water presented medium cancer risks (9.84 × 10^–5^) to children and low-medium cancer risks (1.44 × 10^–5^) to adults. The highest cancer risks for both children (2.24 × 10^–1^) and adults (3.28 × 10^–2^) occurred at sampling site NY-07, implying that 2 in 10 children and 3 in 100 adults have the probability of developing excess cancer in their lifetime due to PEM exposure from the River Nyamwamba water. This cancer risk is horrendous and unacceptable for both children and adults. The cancer risk levels reported in the present study were greater than the values of 5.77 × 10^–6^ for children and 7.07 × 10^–6^ for adults reported in Shanono and Bagwai Artisanal gold mines in Nigeria (Bello et al., 2019).

The cancer risks reported in this study are very conservative estimates due to the use of filtered water samples and the US standard residential exposure parameters adopted in the computation, which may not necessarily be suitable for a typical Ugandan residential exposure scenario (US EPA, 2004). In addition, soil, dust, and food items were not incorporated in this study, yet would be important components of the exposure routes and hence should be considered for future studies. Nonetheless, the calculated risks provide insight into the proper management of water resources.

Even though the cancer risks from Co, Cu, and Mn exposures were not computed due to the nonavailability of the respective cancer slope factors, the long-term exposures to these PEMs may result in various adverse effects, including lung fibrosis, hepatotoxicity, neural system disorders, heart diseases, liver damage, anaemia, fatigue, nerve damage, obesity, detrimental impact on male reproductive function, and manganese poisoning in humans (Elnabi et al., 2023; Hameed et al., 2020). The Co, Cu, and Mn concentrations exhibited in the River Nyamwamba water potentially pose a human health threat to the population living within the Kilembe area and areas immediately adjacent downstream.

## Conclusion and recommendations

This study investigated the concentrations of potentially ecotoxic metal(loid)s (PEM), including Al, As, Cd, Co, Cr, Cu, Fe, Mn, Mo, Ni, Pb, and Zn in the mining-affected River Nyamwamba, Uganda. It was shown that mining activities have significantly degraded the water quality through the release of PEMs, particularly Co, Mn, and Ni, which often exceed national and international safety thresholds. The contamination is most severe within the mining area and is primarily driven by mine drainage and leachate from the processing plant and tailings storage facilities. While regulatory standards suggest moderate compliance, comparison with baseline conditions and ecological risk indices reveals extreme contamination and serious ecological threats. Human health risk assessments indicate that exposure to metals (especially through dermal contact) poses significant non-carcinogenic risks to both adults and children, with children being more vulnerable. Moreover, the estimated cancer risks, largely driven by Ni, are high and well beyond acceptable limits, highlighting an urgent need for remedial action. These findings underscore the critical importance of implementing stricter pollution controls, improving waste management at mining sites, and developing location-specific risk models to better protect both environmental and public health in the region.

From the findings of the study, to protect tropical mining-affected aquatic ecosystems and human health, it is recommended that 1) the local government authorities should sensitise the community and restrict the use of River Nyamwamba water for domestic and drinking purposes in favour of the available shallow wells, boreholes, springs, and rainwater harvesting, 2) water discharge into tropical rivers and streams should be treated before release, 3) contaminant sources in tropical aquatic ecosystems should be identified, remediated (if possible) and monitored, and 4) comprehensive studies on PEM contaminated tropical aquatic ecosystems including soils, dust and food items should be considered to understand all exposure routes.

## .

## Supplementary Information

Below is the link to the electronic supplementary material.

Supplementary file1 (DOCX 3518 KB)

Supplementary file2 (DOCX 54 KB)

## Data Availability

Data will be made available on request.
