# Supplementary material for: Aqueous potentially ecotoxic metal(loid)s in a tropical mining-affected river system: sources and environmental and human health risks
Source: Environ Geochem Health. 2025 Oct 17;47(11):504. doi: 10.1007/s10653-025-02808-y (PMC12534356; doi:10.1007/s10653-025-02808-y)
Supplement: Supplementary file 1 — Supplementary file1 (DOCX 3518 KB) [file 10653_2025_2808_MOESM1_ESM.docx]

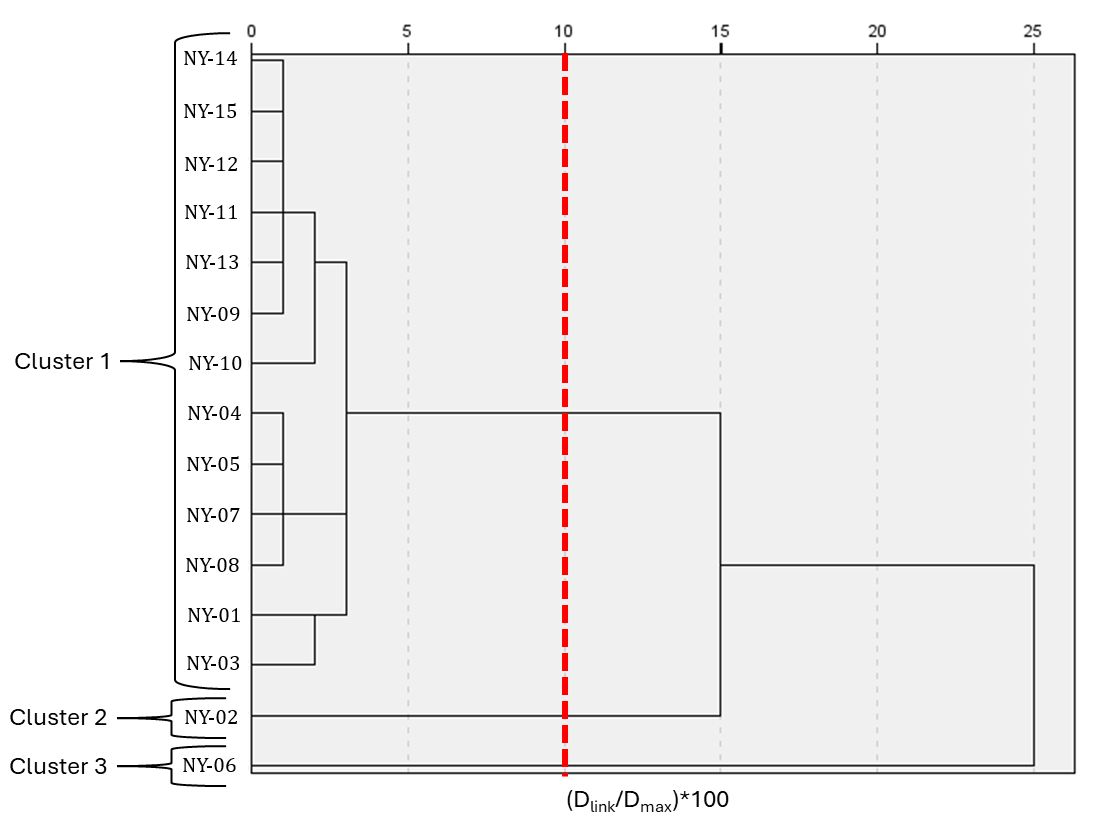


**Fig. S1** Dendrogram showing clustering of the sampling sites using Average Linkage (Between Groups)


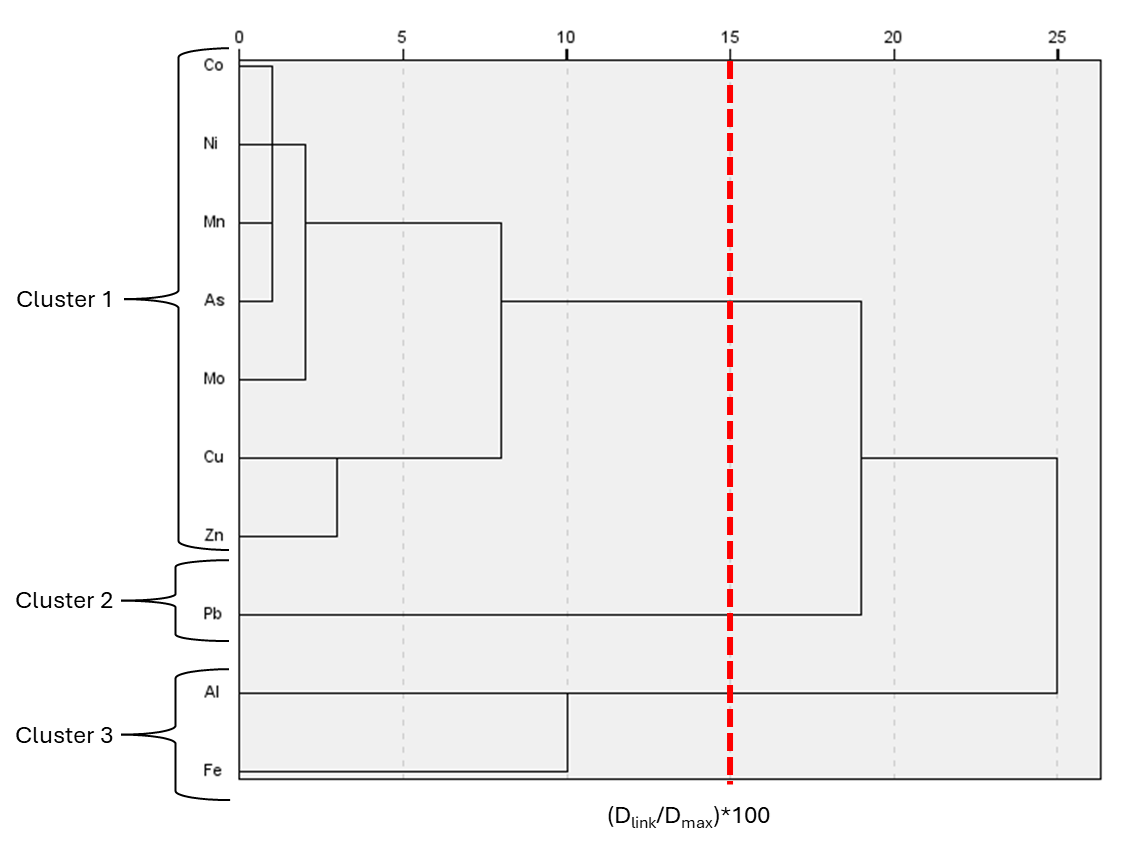


**Fig. S2** Dendrogram showing clustering of the PEMs using Average Linkage (Between Groups)
