# Supplementary material for: Aqueous potentially ecotoxic metal(loid)s in a tropical mining-affected river system: sources and environmental and human health risks
Source: Environ Geochem Health. 2025 Oct 17;47(11):504. doi: 10.1007/s10653-025-02808-y (PMC12534356; doi:10.1007/s10653-025-02808-y)
Supplement: Supplementary file 2 — Supplementary file2 (DOCX 54 KB) [file 10653_2025_2808_MOESM2_ESM.docx]

Table S1. Location of sampling sites

| Sampling site | Description | Zone | UTM EAST (m) | UTM North (m) | Elevation (m) |
| --- | --- | --- | --- | --- | --- |
| NY-01 | Upstream of River Nyamwamba | 36N | 165,997 | 24,319 | 1,749 |
| NY-02 | River Nyarusegi | 36N | 166,744 | 22,605 | 1,612 |
| NY-03 | River Kanyaruboga | 36N | 167,177 | 22,508 | 1,609 |
| NY-04 | River Nyamwamba | 36N | 167,161 | 22,187 | 1,592 |
| NY-05 | River Nyamwamba | 36N | 167,281 | 22,088 | 1,591 |
| NY-06 | Underground Mine Drainage Channel | 36N | 167,276 | 22,025 | 1,598 |
| NY-07 | River Nyamwamba | 36N | 167,786 | 21,589 | 1,482 |
| NY-08 | River Nyamwamba | 36N | 167,944 | 20,945 | 1,456 |
| NY-09 | River Ngangi | 36N | 168,028 | 20,534 | 1,446 |
| NY-10 | River Kyanzusu | 36N | 168,941 | 20,206 | 1,425 |
| NY-11 | River Nyamwamba | 36N | 170,009 | 21,003 | 1,387 |
| NY-12 | River Nyamwamba | 36N | 173,670 | 21,432 | 1,181 |
| NY-13 | River Rukoki | 36N | 173,318 | 21,592 | 1,184 |
| NY-14 | River Nyamwamba | 36N | 174,908 | 21,469 | 1,151 |
| NY-15 | Downstream of River Nyamwamba | 36N | 178,773 | 20,343 | 1,105 |
| LG-01 | Mouth of River Nyamwamba in Lake George | 36N | 185,163 | 7,004 | 1,003 |
| LG-02 | Centre of Lake George | 36N | 192,126 | 10 | 905 |
| LG-03 | Lake George | 36N | 187,559 | - 2,568 | 905 |
| LG-04 | Lake George | 36N | 184,798 | 1,053 | 1,099 |

Table S2. Parameters for human health risk calculations (from US EPA, 2004).

| Parameter | Unit | Child | Adult |
| --- | --- | --- | --- |
| IR | L per day | 1 | 2 |
| ED | years | 6 | 30 |
| EF | days per year | 350 | 350 |
| ET | h | 1 | 0.58 |
| SA | cm^2^ | 6600 | 18000 |
| AT | Days | 2190 | 25600 |
| BW | kg | 15 | 70 |

Table S3. Cancer risk classification categories (from Dashtizadeh et al., 2019).

| Grade | Risk | CR Values | Description |
| --- | --- | --- | --- |
| I | Extremely negligible | CR ≤ 1.00 × 10^-6^ | Completely accept risk |
| II | Negligible | 1.00 × 10^-6^ < CR ≤ 1.00 × 10^-5^ | No need to care about the risk |
| III | Low-medium | 1.00 × 10^-5^ < CR ≤ 5.00 × 10^-5^ | Do not mind about the risk |
| IV | Medium | 5.00 × 10^-5^ < CR ≤ 1.00 × 10^-4^ | Need to care about the risk |
| V | Medium-high | 1.00 × 10^-4^ < CR ≤ 5.00 × 10^-4^ | Need to care about the risk |
| VI | Elevated | 5.00 × 10^-4^ < CR ≤ 1.00 × 10^-3^ | Need attention and immediate action |
| VII | Extremely elevated | CR > 1.00 × 10^-3^ | Must solve it |

Table S4. Hazard quotients (HQ) and hazard index (HI) for PEMs in River Nyamwamba water.

| Sampling Sites | HQ ingestion | | | | | | | | | HQ dermal | | | | | | | | | Total HQing | Total HQderm | HI |
| --- | --- | --- | --- | --- | --- | --- | --- | --- | --- | --- | --- | --- | --- | --- | --- | --- | --- | --- | --- | --- | --- |
|  | As | Co | Cu | Fe | Mn | Mo | Ni | Pb | Zn | As | Co | Cu | Fe | Mn | Mo | Ni | Pb | Zn |  |  |  |
| CHILD | | | | | | | | | | | | | | | | | | | | | |
| NY-01 | 0.01 | 0.00 | 0.03 | 0.01 | 0.01 | 0.01 | 0.00 | 0.01 | 0.00 | 0.04 | 0.05 | 0.35 | 5.60 | 1.32 | 0.05 | 0.01 | 0.04 | 0.00 | 0.07 | 7.47 | 7.54 |
| NY-04 | 0.01 | 1.05 | 2.89 | 0.00 | 0.20 | 0.01 | 0.33 | 0.00 | 0.01 | 0.04 | 927 | 33.5 | 1.65 | 33.4 | 0.04 | 10.9 | 0.02 | 0.21 | 4.51 | 1,007 | 1,012 |
| NY-05 | 0.00 | 1.05 | 2.54 | 0.00 | 0.21 | 0.01 | 0.35 | 0.00 | 0.01 | 0.03 | 925 | 29.4 | 2.23 | 35.2 | 0.04 | 11.5 | 0.02 | 0.22 | 4.18 | 1,003 | 1,008 |
| NY-07 | 0.01 | 1.20 | 2.39 | 0.00 | 0.28 | 0.01 | 0.36 | 0.00 | 0.01 | 0.04 | 1,058 | 27.7 | 1.21 | 45.5 | 0.05 | 11.9 | 0.01 | 0.22 | 4.26 | 1,145 | 1,149 |
| NY-08 | 0.00 | 0.36 | 1.61 | 0.01 | 0.09 | 0.01 | 0.10 | 0.00 | 0.00 | 0.02 | 320 | 18.6 | 4.16 | 15.1 | 0.04 | 3.44 | 0.01 | 0.06 | 2.19 | 361 | 363 |
| NY-11 | 0.00 | 0.41 | 1.24 | 0.02 | 0.11 | 0.01 | 0.10 | 0.00 | 0.00 | 0.02 | 362 | 14.4 | 7.72 | 18.1 | 0.04 | 3.32 | 0.01 | 0.07 | 1.89 | 405 | 407 |
| NY-12 | 0.00 | 0.29 | 1.26 | 0.02 | 0.09 | 0.01 | 0.07 | 0.00 | 0.00 | 0.03 | 257 | 14.6 | 11.7 | 15.4 | 0.04 | 2.40 | 0.01 | 0.04 | 1.76 | 301 | 303 |
| NY-14 | 0.00 | 0.26 | 1.20 | 0.03 | 0.09 | 0.01 | 0.06 | 0.00 | 0.00 | 0.03 | 231 | 13.9 | 11.8 | 14.3 | 0.04 | 2.13 | 0.02 | 0.03 | 1.65 | 273 | 275 |
| NY-15 | 0.00 | 0.18 | 1.06 | 0.03 | 0.08 | 0.01 | 0.05 | 0.00 | 0.00 | 0.03 | 158 | 12.2 | 12.1 | 12.4 | 0.04 | 1.56 | 0.02 | 0.05 | 1.40 | 197 | 198 |
| ADULT | | | | | | | | | | | | | | | | | | | | | |
| NY-01 | 0.00 | 0.00 | 0.01 | 0.00 | 0.00 | 0.00 | 0.00 | 0.00 | 0.00 | 0.01 | 0.01 | 0.05 | 0.81 | 0.19 | 0.01 | 0.00 | 0.01 | 0.00 | 0.01 | 1.08 | 1.10 |
| NY-04 | 0.00 | 0.19 | 0.53 | 0.00 | 0.04 | 0.00 | 0.06 | 0.00 | 0.00 | 0.01 | 135 | 4.87 | 0.24 | 4.85 | 0.01 | 1.59 | 0.00 | 0.03 | 0.83 | 146 | 147 |
| NY-05 | 0.00 | 0.19 | 0.47 | 0.00 | 0.04 | 0.00 | 0.06 | 0.00 | 0.00 | 0.00 | 134 | 4.27 | 0.32 | 5.11 | 0.01 | 1.67 | 0.00 | 0.03 | 0.77 | 146 | 147 |
| NY-07 | 0.00 | 0.22 | 0.44 | 0.00 | 0.05 | 0.00 | 0.07 | 0.00 | 0.00 | 0.01 | 154 | 4.02 | 0.18 | 6.61 | 0.01 | 1.74 | 0.00 | 0.03 | 0.78 | 166 | 167 |
| NY-08 | 0.00 | 0.07 | 0.30 | 0.00 | 0.02 | 0.00 | 0.02 | 0.00 | 0.00 | 0.00 | 46.5 | 2.70 | 0.60 | 2.19 | 0.01 | 0.50 | 0.00 | 0.01 | 0.40 | 52.5 | 52.9 |
| NY-11 | 0.00 | 0.08 | 0.23 | 0.00 | 0.02 | 0.00 | 0.02 | 0.00 | 0.00 | 0.00 | 52.5 | 2.09 | 1.12 | 2.62 | 0.01 | 0.48 | 0.00 | 0.01 | 0.35 | 58.9 | 59.2 |
| NY-12 | 0.00 | 0.05 | 0.23 | 0.00 | 0.02 | 0.00 | 0.01 | 0.00 | 0.00 | 0.00 | 37.3 | 2.12 | 1.70 | 2.24 | 0.01 | 0.35 | 0.00 | 0.01 | 0.32 | 43.7 | 44.0 |
| NY-14 | 0.00 | 0.05 | 0.22 | 0.00 | 0.02 | 0.00 | 0.01 | 0.00 | 0.00 | 0.00 | 33.5 | 2.02 | 1.72 | 2.08 | 0.01 | 0.31 | 0.00 | 0.00 | 0.30 | 39.7 | 40.0 |
| NY-15 | 0.00 | 0.03 | 0.19 | 0.00 | 0.01 | 0.00 | 0.01 | 0.00 | 0.00 | 0.00 | 23.0 | 1.78 | 1.76 | 1.81 | 0.01 | 0.23 | 0.00 | 0.01 | 0.26 | 28.5 | 28.8 |

Table S5. Cancer risks (CR) for PEMs in River Nyamwamba water.

| Sampling Sites | ELCR Ingestion | | | CR ingestion | ELCR Dermal | | | CR dermal | CR | | | Total CR |
| --- | --- | --- | --- | --- | --- | --- | --- | --- | --- | --- | --- | --- |
|  | As | Ni | Pb |  | As | Ni | Pb |  | As | Ni | Pb |  |
| CHILD | | | | | | | | | | | | |
| NY-01 | 2.43E-05 | 2.85E-06 | 2.25E-07 | 3.08E-06 | 1.69E-04 | 9.41E-05 | 1.27E-06 | 9.53E-05 | 1.93E-04 | 9.69E-05 | 1.49E-06 | 2.91E-04 |
| NY-04 | 2.47E-05 | 6.02E-03 | 8.03E-08 | 6.02E-03 | 1.72E-04 | 1.99E-01 | 4.53E-07 | 1.99E-01 | 1.97E-04 | 2.05E-01 | 5.34E-07 | 2.05E-01 |
| NY-05 | 2.16E-05 | 6.35E-03 | 1.04E-07 | 6.35E-03 | 1.50E-04 | 2.10E-01 | 5.86E-07 | 2.10E-01 | 1.72E-04 | 2.16E-01 | 6.90E-07 | 2.16E-01 |
| NY-07 | 2.68E-05 | 6.59E-03 | 6.10E-08 | 6.59E-03 | 1.86E-04 | 2.17E-01 | 3.44E-07 | 2.17E-01 | 2.13E-04 | 2.24E-01 | 4.05E-07 | 2.24E-01 |
| NY-08 | 1.40E-05 | 1.90E-03 | 3.70E-08 | 1.90E-03 | 9.74E-05 | 6.25E-02 | 2.08E-07 | 6.25E-02 | 1.11E-04 | 6.44E-02 | 2.45E-07 | 6.45E-02 |
| NY-11 | 1.13E-05 | 1.83E-03 | 7.41E-08 | 1.83E-03 | 7.84E-05 | 6.03E-02 | 4.18E-07 | 6.03E-02 | 8.96E-05 | 6.22E-02 | 4.92E-07 | 6.23E-02 |
| NY-12 | 1.71E-05 | 1.32E-03 | 7.18E-08 | 1.33E-03 | 1.19E-04 | 4.37E-02 | 4.05E-07 | 4.37E-02 | 1.36E-04 | 4.50E-02 | 4.77E-07 | 4.52E-02 |
| NY-14 | 1.99E-05 | 1.17E-03 | 9.14E-08 | 1.17E-03 | 1.38E-04 | 3.87E-02 | 5.15E-07 | 3.87E-02 | 1.58E-04 | 3.99E-02 | 6.07E-07 | 4.00E-02 |
| NY-15 | 1.85E-05 | 8.62E-04 | 9.03E-08 | 8.62E-04 | 1.28E-04 | 2.84E-02 | 5.09E-07 | 2.84E-02 | 1.47E-04 | 2.93E-02 | 6.00E-07 | 2.95E-02 |
| ADULT | | | | | | | | | | | | |
| NY-01 | 4.46E-06 | 5.24E-07 | 4.12E-08 | 5.65E-07 | 2.45E-05 | 1.37E-05 | 1.84E-07 | 1.38E-05 | 2.89E-05 | 1.42E-05 | 2.25E-07 | 4.33E-05 |
| NY-04 | 4.55E-06 | 1.11E-03 | 1.48E-08 | 1.11E-03 | 2.50E-05 | 2.89E-02 | 6.58E-08 | 2.89E-02 | 2.95E-05 | 3.00E-02 | 8.06E-08 | 3.00E-02 |
| NY-05 | 3.98E-06 | 1.17E-03 | 1.91E-08 | 1.17E-03 | 2.18E-05 | 3.04E-02 | 8.52E-08 | 3.04E-02 | 2.58E-05 | 3.16E-02 | 1.04E-07 | 3.16E-02 |
| NY-07 | 4.93E-06 | 1.21E-03 | 1.12E-08 | 1.21E-03 | 2.71E-05 | 3.16E-02 | 5.00E-08 | 3.16E-02 | 3.20E-05 | 3.28E-02 | 6.12E-08 | 3.28E-02 |
| NY-08 | 2.57E-06 | 3.48E-04 | 6.79E-09 | 3.48E-04 | 1.41E-05 | 9.09E-03 | 3.03E-08 | 9.09E-03 | 1.67E-05 | 9.43E-03 | 3.71E-08 | 9.45E-03 |
| NY-11 | 2.07E-06 | 3.36E-04 | 1.36E-08 | 3.36E-04 | 1.14E-05 | 8.76E-03 | 6.08E-08 | 8.77E-03 | 1.35E-05 | 9.10E-03 | 7.44E-08 | 9.11E-03 |
| NY-12 | 3.13E-06 | 2.43E-04 | 1.32E-08 | 2.43E-04 | 1.72E-05 | 6.35E-03 | 5.89E-08 | 6.35E-03 | 2.04E-05 | 6.60E-03 | 7.21E-08 | 6.62E-03 |
| NY-14 | 3.66E-06 | 2.15E-04 | 1.68E-08 | 2.15E-04 | 2.01E-05 | 5.62E-03 | 7.49E-08 | 5.62E-03 | 2.38E-05 | 5.83E-03 | 9.17E-08 | 5.86E-03 |
| NY-15 | 3.39E-06 | 1.58E-04 | 1.66E-08 | 1.58E-04 | 1.86E-05 | 4.13E-03 | 7.40E-08 | 4.13E-03 | 2.20E-05 | 4.29E-03 | 9.06E-08 | 4.31E-03 |
